# Dynamics of Colonization and Expression of Pathogenicity Related Genes in *Fusarium oxysporum* f.sp. *ciceri* during Chickpea Vascular Wilt Disease Progression

**DOI:** 10.1371/journal.pone.0156490

**Published:** 2016-05-26

**Authors:** Medha L. Upasani, Gayatri S. Gurjar, Narendra Y. Kadoo, Vidya S. Gupta

**Affiliations:** Biochemical Sciences Division, CSIR-National Chemical Laboratory, Dr. Homi Bhabha Road, Pashan, Pune 411008, India; Universita degli Studi di Pisa, ITALY

## Abstract

Fusarium wilt caused by *Fusarium oxysporum* f.sp. *ciceri* (Foc) is a constant threat to chickpea productivity in several parts of the world. Understanding the molecular basis of chickpea-Foc interaction is necessary to improve chickpea resistance to Foc and thereby the productivity of chickpea. We transformed Foc race 2 using green fluorescent protein (GFP) gene and used it to characterize pathogen progression and colonization in wilt-susceptible (JG62) and wilt-resistant (Digvijay) chickpea cultivars using confocal microscopy. We also employed quantitative PCR (qPCR) to estimate the pathogen load and progression across various tissues of both the chickpea cultivars during the course of the disease. Additionally, the expression of several candidate pathogen virulence genes was analyzed using quantitative reverse transcriptase PCR (qRT-PCR), which showed their characteristic expression in wilt-susceptible and resistant chickpea cultivars. Our results suggest that the pathogen colonizes the susceptible cultivar defeating its defense; however, albeit its entry in the resistant plant, further proliferation is severely restricted providing an evidence of efficient defense mechanism in the resistant chickpea cultivar.

## Introduction

Chickpea (*Cicer arietinum* L.) ranks third after common bean (*Phaseolus vulgaris*) and peas (*Pisum sativum*) in grain legume production and is the only large-scale cultivated crop within the *Cicer* genus [1]. It is grown in over 50 countries with 90% of its area in developing countries. Chickpea yield has been mostly stagnant over the years due to its susceptibility to various biotic and abiotic factors. The important biotic factors affecting chickpea productivity include Fusarium wilt caused by *Fusarium oxysporum* f.sp. *ciceri* in the tropics and Ascochyta blight caused by *Ascochyta rabii* in temperate regions [2]. Eight races have been reported for *Fusarium oxysporum* f.sp. *ciceri* (Foc), worldwide out of which six (1A, 2, 3, 4, 5 and 6) cause wilting; whereas the races 0 and 1B/C cause yellowing symptoms [3, 4]. The pathogen can survive in soils for up to six years even without the host, which makes its control very difficult [5]. Conventional strategies, such as crop rotations, avoiding the infected field to grow chickpea and the use of chemical fungicides are being used to manage the disease. However, they have not been successful in controlling the disease [6].

Plant-pathogen interaction is complex and involves the expression of both, pathogen virulence genes as well as plant defense genes. Till date, various candidate genes with prime role in fungal pathogenesis have been identified [7]. These fungal pathogenicity genes are categorized based on formation of infection structures, cell wall degradation, toxin biosynthesis, signaling and proteins suppressing plant defense [8–10]. In *F*. *oxysporum*, cell wall degrading enzymes (CWDE), involved in penetration and colonization in the host plant have been studied. Pectate lyase, a CWDE, has been suggested as a Foc pathogenicity factor in chickpea wilt [11]. Signaling genes expressed during pathogenesis such as *fmk1* (a mitogen-activated protein kinase) in *F*. *oxysporum* f.sp. *lycopersici* [12] and G protein subunits including G protein α subunit (fga1) and β subunit (fgb1) in *F*. *oxysporum* f.sp. *cucumerinum* have reported to be necessary for fungal morphogenesis, development and virulence [13, 14]. In addition, the genes *Fow*1 and *Fow*2 [15, 16], *Six*1, *Six*2 and *Six*3 [17] and Frp1 F-box protein [18] in *F*. *oxysporum* f.sp. *lycopersici* (Fol) have been suggested to play essential role during pathogenesis in tomato.

In the present study, we transformed Foc race 2 with the *eGFP* gene encoding green florescent protein (GFP) and used it to understand the infection process and colonization patterns in wilt-susceptible and wilt-resistant chickpea cultivars. Quantitative PCR (qPCR) revealed significant differences in pathogen load in both the cultivars. The expression of several pathogen virulence related genes involved in processes like signaling, cell wall degradation and fungal morphogenesis, as well as those identified in previous studies to be important for fungal pathogenesis was also analyzed. All the three approaches revealed similar and significant differences among the wilt-susceptible and wilt-resistant chickpea cultivars.

## Materials and Methods

### Plant material, fungal culture and inoculation

Seeds of chickpea cultivars JG62 and Digvijay were obtained from Mahatma Phule Krishi Vidyapeeth (MPKV), Rahuri, India and the culture of *Fusarium oxysporum* f.sp. *ciceri* standard race 2 (NRRL 32154) (Foc 2) was obtained from the International Crops Research Institute for Semi-Arid Tropics (ICRISAT), Patancheru, Andhra Pradesh, India. JG62 (selection from germplasm) is susceptible to Fusarium wilt, while Digvijay (Phule G-91028 X Bheema) is resistant to the disease [7]. The seeds were surface-sterilized using 1% sodium hypochlorite and soaked overnight in sterile deionized water. They were wrapped in wet sterile muslin cloth till sprouting and transferred to surface-sterilized plastic cups containing autoclaved Soil Rite (mixture of 75% Irish Peatmoss and 25% horticulture grade Expanded Perlite; obtained from M/s Naik Krushi Udyog, Pune, India). The plants were grown for one week in growth chamber (14 h light/10 h dark, 22–25°C, 50–60% relative humidity) and inoculated by root clipping [19] with freshly prepared spore suspension (1 × 10^6^ spores/ml) of Foc 2. Root tips of tap root and lateral roots were cut and the entire root system was dipped in spore suspension for 5 min. Plants mock-inoculated with sterile deionized water served as controls. Thus four treatments *viz*. JG62 inoculated (JGI); JG62 control (JGC); Digvijay inoculated (DVI), and Digvijay control (DVC); comprising 10 plants per time-point at eight different time-points, *viz*. 0 hpi, 16 hpi, 24 hpi (hours post inoculation), 2 dpi, 4 dpi, 7 dpi, 14 dpi and 28 dpi (days post inoculation) in three replicates each were raised in growth chamber. The plants were lightly watered using autoclaved tap water every 2–3 days.

### Evaluation of disease symptoms and tissue collection

The plants were evaluated for morphological changes and development of wilting symptoms daily after inoculation. Tissues of all the four treatments were collected at all the eight time points mentioned above. The time scale of the infection process was divided as: a) early stage (0 hpi to 7 dpi), b) middle stage (7 to 14 dpi), and c) late stage (14 to 28 dpi) based on the morphological symptoms observed. Two types of tissues were collected: whole roots and root fractions of approximately 2 inches in length, as well as two fractions of shoot till 2^nd^ internode. The tissues were flash frozen in liquid nitrogen and stored at -80°C till further analysis.

### *eGFP* transformation of Foc 2

A kill curve was initially set up for Foc using the poisoned food technique [20] to determine the minimum inhibitory concentration of hygromycin B. For this, potato dextrose agar (PDA) was supplemented with 25, 50, 75 and 100 μg/ml of hygromycin B and the fungus was grown at 28°C for up to 21 days in dark in triplicates. For transformation of Foc 2, the pathogen was grown in potato dextrose broth (PDB) without hygromycin B at 26–28°C for 10 days with shaking at 180 rpm and conidia were harvested in sterile water. Spore count was recorded using a haemocytometer [21]. The LBA4404 strain of *Agrobacterium tumefaciens* containing modified pCAMBIA 1302 (mGFP cassette replaced with eGFP cassette from pCBdeltaXCE) was used for transformation [22]. To express *eGFP* in the fungus, a cassette containing *eGFP* with *citA* promoter in plasmid pCBdeltaXCE was amplified with a restriction site for *Bst*EII (G’GTGACC) at the start of the *eGFP* gene [22]. This cassette and pCAMBIA 1302 were then double digested with *Xba*I (T’CTAGA) and *Bst*EII (G’GTGACC) and the sticky end products were ligated using T_4_ DNA ligase. Thus, a modified pCAMBIA 1302 vector with *eGFP* gene and *citA* promoter was constructed and used to transform Foc 2 (**S1 Fig**).

Foc 2 transformation was performed according to Mullins et al [23] with some modifications [24, 25]. An overnight grown single colony of *A*. *tumefaciens* LBA4404 was transferred to a flask containing minimal medium supplemented with rifampicin (50μg/ml) and kanamycin (50μg/ml) for two days at 25°C with shaking at 180 rpm. At 0.600 OD (600λ), the cells were diluted in induction medium containing 200 μM acetosyringone and were allowed to grow for an additional 5–7 hrs at 26–27°C at 200 rpm. Microconidia (1.0×10^6^/ml) from a 10 days old Foc 2 culture were mixed in equal proportion with acetosyringone-induced *A*. *tumefaciens* cells (0.300 OD), incubated for 10 min at 22°C and plated on Hybond N+ membrane spread over the co-cultivation medium [26] containing 200 μM acetosyringone. The plates were incubated at 23°C for 48 to 60 hr. The membranes were transferred to PDA selection medium containing 100 μg/ml hygromycin B and 200 μM cefotaxime, the latter antibiotic was used to kill *Agrobacterium*. Hygromycin B resistant (HygR) putatively transformed colonies of Foc 2 were observed following 7–9 days of incubation at 23–25°C. These colonies were serially transferred five times in the selection medium to confirm stability of transformation, growth and morphology. Hygromycin resistant single conidial cultures of the transformants were preserved in 25% glycerol at −80°C for long term storage and analysis.

### Phenotypic characterization of wild type and transformed Foc 2

The Foc 2 transformants were transferred to PDA (containing hygromycin B) to observe colony morphology and cultural characteristics with respect to wild-type. Slide preparations using a drop of sterile water and hyphae were done for both the wild-type and the transformants. Mycelial growth rate of both was evaluated in triplicate by placing a PDA plug of actively growing culture on PDA plate and incubated at 26–28°C. The radial mycelial growth (RMG) was determined by measuring the length of four radii (each radius in one direction) daily. The radial growth rate (RGR) was calculated by the slope of the linear regression of the mean colony radius over time [27]. In addition, pathogenicity of the transformants was evaluated in comparison to the wild type. Microconidial suspensions of wild-type Foc 2 and five transformants were used to inoculate the susceptible (JG62) and resistant (Digvijay) chickpea cultivars as described earlier. Three replicates of five seedlings per cultivar per transformant as well as the wild-type were inoculated. The seedlings mock inoculated with sterile deionized water served as control.

### Measurement of GFP fluorescence

GFP fluorescence in five selected *eGFP* transformed Foc 2 isolates was measured using luminescence spectrometer LS-5 (PerkinElmer, USA). The transformants were grown in triplicate at 26–28°C for 72 hr in 10 ml PDB culture at 180 rpm. The cultures were centrifuged at 4000 rpm and the pelleted mycelia were crushed under liquid nitrogen and transferred to 5 ml extraction buffer (10 mM Tris pH 7.4, 1 mM CaCl_2_). Mycelial debris was removed by another round of centrifugation. The resulting supernatant was assayed for fluorescence using 488 nm and 512 nm wavelengths for excitation and emission, respectively. Protein concentration was measured using Bradford assay with bovine serum albumin as standard [28, 29]. Relative fluorescence units (RFU) values were then normalized with respect to protein concentration.

### Molecular characterization of *eGFP* transformed Foc 2

Five selected *eGFP* transformed Foc 2 isolates were grown in 100 ml PDB containing hygromycin B (75 μg/ml) at 28°C and 180 rpm for 4–5 days. Mycelial mass collected by filtration through muslin cloth was crushed to fine powder under liquid nitrogen and DNA was isolated using modified CTAB protocol [30]. PCR was used to confirm the presence of hygromycin (*hph*) and *eGFP* genes in transformed isolates. Both transformed as well as wild-type Foc 2 DNA were used for PCR amplification with *hph* and *eGFP* specific primers *viz*. Hph F (5’-TCCTGCAAGCTCCGGATGCCC-3’) and Hph R (5’-CGTGCACAGGGTGTCACGTTGC-3’); hph new F (5’-CTCGGACGAGTGCTGGGGCGT-3’) and hph new R (5’-AAGCCTGAACTCACCGCGACGTCTG-3’); eGFP1F (5’-ACGTAAACGGCCACAAGTTC-3’) and eGFP1R (5’TGCTCAGGTAGTGGTTGTCG-3’) to confirm the presence of *hph* and *eGFP* genes in the transformants.

### Microscopic monitoring of pathogen progression in chickpea plants

Another set of JG62 and Digvijay plants was inoculated with the selected transformant (D4). The inoculated and control chickpea plants were sampled daily during 1 to 4 DPI and at a 2–3 day interval thereafter, up to 18 DPI. During each sampling, four plants were collected from each treatment. The entire surface of the tap and lateral roots of each plant was observed under a confocal laser scanning microscope (CLSM). Images were acquired by excitation with 488 nm argon laser (515–530 nm) for detection of fluorescence emitted by the pathogen using a Zeiss™ 710 CLSM system (Carl Zeiss Inc., USA). In addition, auto fluorescence of chickpea plants was assessed at wavelengths of 550–590 nm. The images were observed using a Zeiss Axio Observer™ inverted microscope with a 20×1.3 NA Plan-Apochromat objective.

### *In planta* pathogen quantification

Three sets of primers *viz*. IV-SP & IV-ASP; Foc 1F & Foc 1R and Foc 3F & Foc 3R were designed to specifically amplify an internal portion of the 1.5-kb sequence characterized amplified region (SCAR) (GenBank accession no. AF492451) of *F*. *oxysporum* f.sp. *ciceri* [31] using Primer3Plus (http://www.bioinformatics.nl/cgi-bin/primer3plus/primer3plus.cgi). PCR conditions were optimized for each primer pair and all the reactions were performed at least twice. Also a positive control (Foc 2 DNA) and negative controls (DNA from un-inoculated chickpea root and no template DNA) were included. The amplification products were electrophoresed and visualized using a gel documentation system (Syngene, USA). The primer combination finally selected was Foc 3F & Foc 3R which amplified an 88-bp fragment (**Table 1**).

**Table 1:** Primer sequences specific to *Fusarium oxysporum* f.sp. *ciceri* 1.5-kb sequence characterized amplified region (SCAR) (GenBank accession no. AF492451) used for quantification of the pathogen in chickpea roots using qRT-PCR.

Primer Name	Sequence (5’ - 3’)	Tann^a^	Amplicon size	Tm^b^
IV-SP	TACGGTACCAGATCATGGCGT	60°C	160 bp	-
IV-ASP	CGCTTTCGATCGTGGCTATG	60°C		
Foc 1F	CATTCGATTCAGGCAAACCT	60°C	88 bp	75.3°C
Foc 1R	TTTCGACCTACGCCAACTCT	60°C		
Foc 3F	AAATGACTGCACCCATGAGAAA	60°C	88 bp	74.9°C
Foc 3R	TGAACCGTAGACCGGAAGGA	60°C		

^a^Annealing temperature (°C)

^b^Melting temperature (°C) at which a specific dissociation peak of increased fluorescence is generated in the melting curve analysis.

To generate standard curves for qPCR assays, 10-fold dilutions of Foc 2 DNA (10 ng/μl) were prepared (1:1, 1:10, 1:10^2^, 1:10^3^, 1:10^4^ and 1:10^5^) in sterile deionized water. The samples were amplified in triplicate using Foc 3F & 3R primers and three independent standard curves were established (**S2 Fig**). To determine pathogen load in susceptible (JG62) and resistant (Digvijay) chickpea plants, genomic DNA isolated from whole roots of inoculated plants was used as template for qPCR. Non-template control as well as DNA from un-inoculated chickpea root served as negative controls. All qPCR amplifications were performed in triplicate using FastStart Universal SYBR Green Master mix (Roche, Germany) and 7900HT Fast Real-Time PCR System (Applied Biosystems, USA). Each reaction contained 30 ng DNA, 0.33 μM of each primer and 15 μl SYBR Green master mix in 30 μl reaction. DNA concentration was adjusted to 10 ng/μl before use in qPCR. The thermocycling profile consisted of initial denaturation at 95°C for 10 min followed by 40 cycles of 95°C for 3 sec and 60°C for 30 sec. Following amplification, a melting dissociation curve was generated using a 60–95°C ramp in order to monitor the specificity of the primers. The exponential phase of the reaction was identified by plotting the fluorescence on a log scale and linear regression analysis was performed to estimate the efficiency of each reaction using Linreg software [32]. The amount of pathogen DNA was estimated in the roots of inoculated cultivars at different time points from the standard curves established earlier. This revealed the biomass of the pathogen at respective time-points. Similar procedure was followed for estimating the biomass of the pathogen in the fractions of chickpea root and shoot tissues (2 inch fractions from the root tip clipped for inoculation) at different time points. The lowest 2 inch fraction of the root was named as R1, while the topmost 2 inch fraction was named as R5. Similarly, S1 was the lowest shoot fraction, followed by S2. JG62 has very short root-length and root mass compared to Digvijay. Hence, the fraction R5 was absent in JG62, while R4 could be collected 7 DPI onwards. Whereas, in Digvijay R4 could be collected from 2 DPI onwards and R5 only at 28 DPI.

### Analysis of expression of Foc 2 virulence related genes

To analyze the expression of virulence related genes of Foc 2 *in planta*, total RNA was isolated from 100 mg ground chickpea root tissue from inoculated plants using the Spectrum Plant Total RNA isolation kit (Sigma-Aldrich, USA), followed by treatment with RNase-free DNase. 1 μg total RNA was reverse transcribed using a high capacity cDNA reverse transcription kit (Sigma-Aldrich, USA). For candidate gene expression analysis, gene specific primers were designed (**Table 2**) from the conserved regions of fungal virulence related genes using the sequences available in NCBI database (database-fungi) (http://www.ncbi.nlm.nih.gov). The specificity of the primers was determined by NCBI Primer BLAST. The elongation factor 1 alpha (*EF1α*) was used as a reference gene. *EF1α* primers were designed to specifically amplify the Foc 2 cDNA and not the chickpea cDNA (**S3 Fig**). qRT-PCR was performed as described earlier and the data were analyzed using the 2^-ΔΔ^Ct method [33].

**Table 2:** Primer sequences of the virulence related genes and *EF1α* (used as a reference gene) used for qRT-PCR.

Target gene	Forward primer sequence (5’ to 3’)	Reverse primer sequence (5’ to 3’)	Amplicon size
Chitin synthase	GGCACAAGGATGAACAACTGGGA	GGAGCTGGATTGAGCATAGGGATC	99 bp
Cell wall protein	GGCAAGCCTTACACCATCCGCTAC	TGTAGTCAGAGAGATCATCGGAGG	91 bp
Mitochondrial carrier protein	CCGCGTTGAGATGCAGAGCAAGAA	GACGCCGTTGGTCTCGTAAATGTAC	97 bp
Glucanosyltransferase	GGCTACATCTGCGGCCAAGACAAG	GAGCACATGCTGTAGGCACCATAG	89 bp
G protein β subunit	GGTCGACCGATAGGAGGCACC	GTGGACCTTGTTGGTGGTATAGGC	86 bp
Xylanase	CCGGCGACGATGTGATGCGA	CCCAGGTGTGGTTGCTCGCT	82 bp
Pectate lyase	GCGGTGCTTTCCATGCTAGCG	GACGAGCTTTCCGTAGTCTTCGGC	98 bp
Polygalacturonase	CTCGCCACTCGACTTGACCTGG	TGAAGCTGTGGTCTGCCCAGTAG	101 bp
*EF1α*	AGCTCGGTAAGGGTTCCTTC	TCCAGAGAGCAATATCGATGG	93 bp

## Results

### Effect of Foc 2 inoculation on susceptible and resistant chickpea cultivars

Seven days old plants of wilt-resistant (Digvijay) and-susceptible (JG62) chickpea cultivars were individually inoculated with fungal spores (DVI and JGI, respectively); while the control plants were mock-inoculated using sterile deionized water (DVC and JGC, respectively). Wilting symptoms started to appear at about 7 dpi in JGI and intensified with time. More than 90% of JGI plants were almost dead by 28 dpi, while the remaining plants wilted severely. On the contrary, all the DVI plants were healthy even beyond 28 dpi (**S4 Fig**) and till maturity. Similarly, the control plants of both the cultivars (JGC and DVC) were healthy throughout the experimental period.

### Generation and characterization of transformants

The minimum inhibitory concentration of hygromycin B for wild-type Foc 2 was 75μg/ml. Hence, the stability of *eGFP* Foc 2 transformants was confirmed by serially transferring them five times to fresh selection medium containing 75μg/ml of hygromycin B. The presence of hygromycin B phophotransferase (*hph*) and *eGFP* genes in the transformants was confirmed by PCR amplicons of sizes 495 bp and 1 kb for *hph* and 546 bp for *eGFP*, respectively; while no amplification was observed in wild-type Foc 2 (**S5 Fig**). The five transformants and the wild-type Foc 2 also showed nearly similar final radial mycelial growth (RMG_F_) and radial growth rate (RGR) values (**Table 3**). No morphological changes in size or shape of vegetative structures were observed. The transformants retained the colony morphology characteristics of the wild-type including white cottony growth of aerial mycelia. Similarly, virulence of all the five transformants was comparable to that of the wild type. The level of GFP fluorescence in the five transformants was variable, while the wild-type Foc 2 showed negligible fluorescence. The transformant ‘D4’ showed the highest fluorescence (60 RFU/mg of protein) (**Table 3**), hence was chosen for further studies.

**Table 3:** Phenotypic characterization of *eGFP* transformants of Foc 2.

*F*. *oxysporum* f.sp. *ciceri* race 2 Wild type and Transformant	Mycelial growth		RFU/μg of protein
	RMG_F_ (mm)	RGR (mm/h)	
Wild type	20.2	0.15	2.11±0.06
A1	18.9	0.13	40.06±0.01
A3	19.3	0.13	18.94±0.01
C2	19.4	0.13	20.12±0.097
C4	19.1	0.13	49.45±0.14
D4	18.7	0.13	60.84±0.17

**A1, A3, C2, C4 and D4** are the isolates transformed with the *eGFP* gene

**RMG**_**F**_: Radial mycelial growth final, assessed by the average value of the fungal colony radius reached after 9 days growth at 25°C under light.

**RGR**: Radial growth rate; calculated by slope of linear regression of the mean colony radius over time. Each value is the mean of three replicates (petri dishes).

**RFU**: Relative fluorescence units.

### Microscopic evaluation of pathogen infection in chickpea cultivars

**Fig 1:**
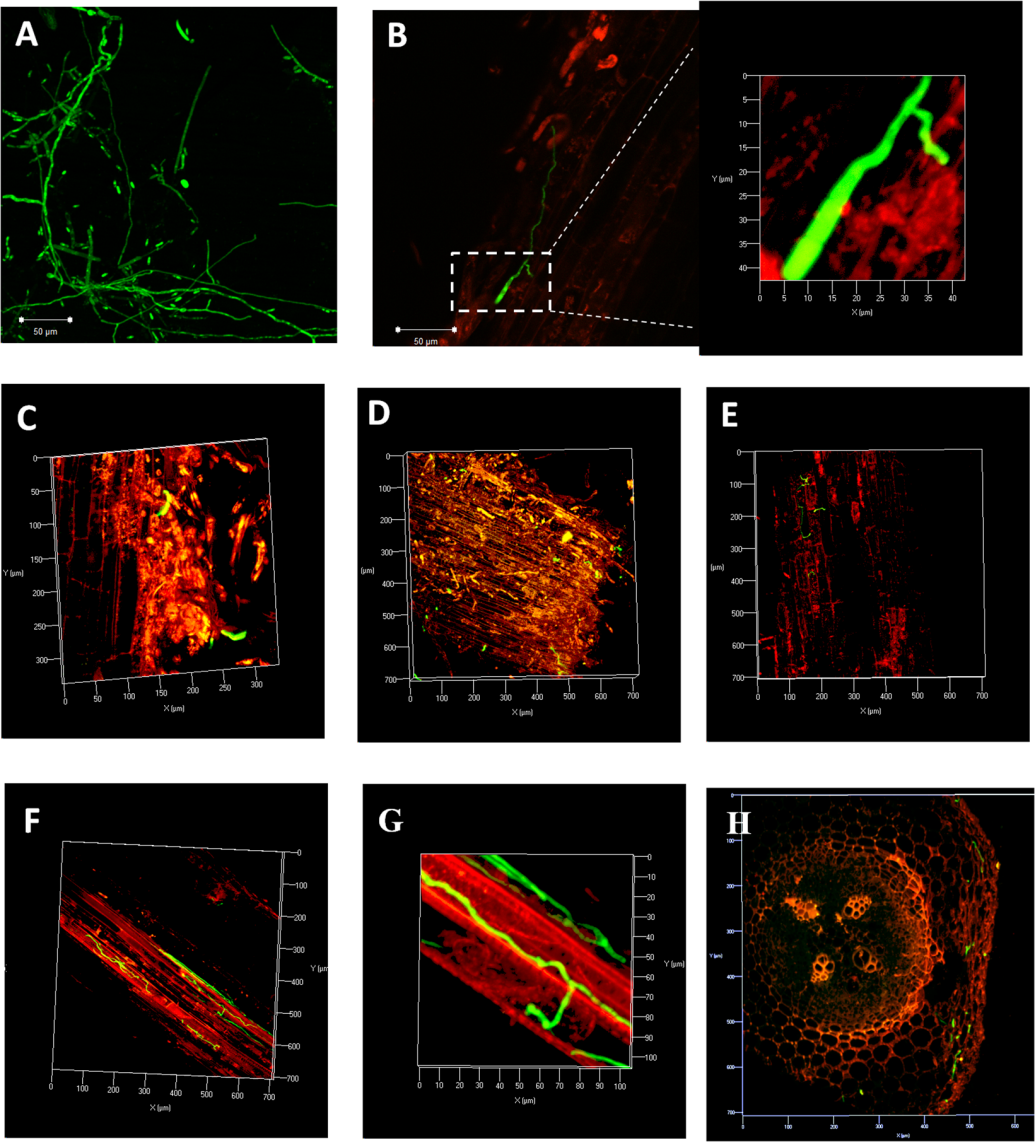
Early stages of chickpea root colonization by Foc 2 marked with eGFP in susceptible (JG62) cultivar by Confocal Laser Scanning Microscopy. **A.** Uniform expression of eGFP in hyphae and spores of transformed isolate D4. **B.** Germinating conidium with primary mycelium in contact with root apex at 24 hpi. **C-D.** Initial hyphal colonization at lower root zone at 2 dpi. **E.** Intermediate root zone showing hyphal colonization extending from epidermis to cortical cells at 2 dpi. **F-G.** Vascular region of root getting colonized at 3 dpi. **H.** Fungal colonization in cortex region of DVI.

Uniform green fluorescence was observed in mycelia and microconidia of the isolate D4 under Confocal Laser Scanning Microscope (CLSM) (**Fig 1A**). Pilot experiments were performed to study the colonization patterns of D4 in JGI and DVI roots. In early stage of infection, colonization on root surface was observed in both the cultivars by forming the primary mycelia at the root apex (**Fig 1B–1D**). Surface colonization was followed by direct penetration of hyphae into epidermal cells without forming any specialized structures (**Fig 1B Inset**). The pathogen then entered root cortex region by 2 dpi (**Fig 1E**). It readily reached the vascular region of lower roots of JGI within 1–3 dpi (**Fig 1F and 1G**), whereas it remained restricted within the cortex region in DVI (**Fig 1H**).

**Fig 2:**
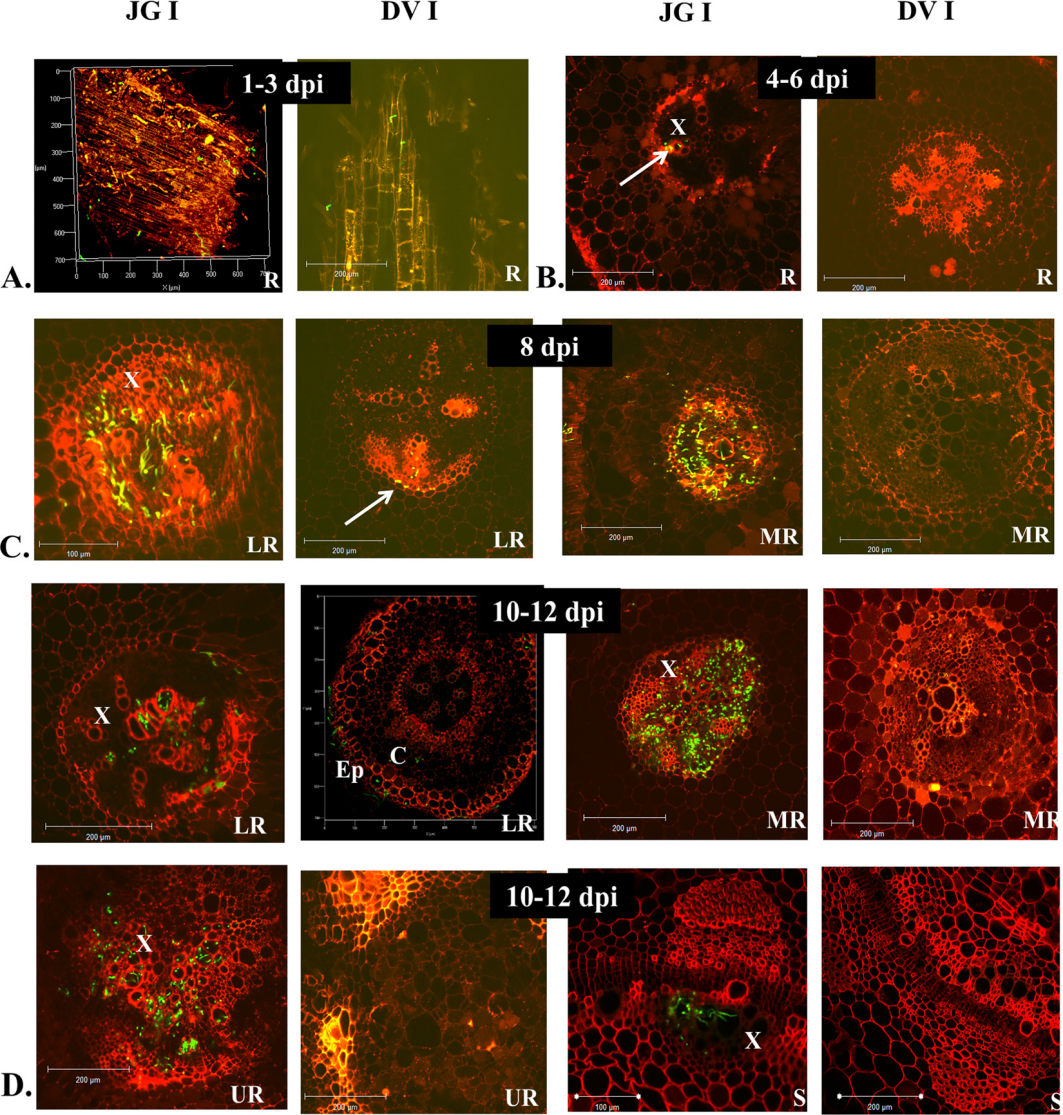
Temporal pattern of colonization of Foc 2 in both susceptible (JG62) and resistant (Digvijay) cultivars of chickpea. R- Root, S- Shoot, LR- Lower Root, MR- Middle Root, UR- Upper Root. **A.** (1–3 dpi) Surface colonization in both susceptible and resistant cultivars. **B.** (4–6 dpi) Transverse sections (TS) of root depicting entry of fungus in vascular bundle only in JGI. **C.** (8 dpi) Transverse sections of lower and middle roots showing increasing amount of fungus in vasculature of JGI while only few fungal mass seen in DVI only in LR. **D.** (10–12 dpi) Transverse sections of all lower, middle, upper roots of JGI reveal higher fungal mass in vascular tissue with few fungal mycelia reaching stem also. However, little fungal mass can be seen till cortex region of TS of LR in DVI with remaining TSs of MR, UR and Stem are clear.

**Fig 3:**
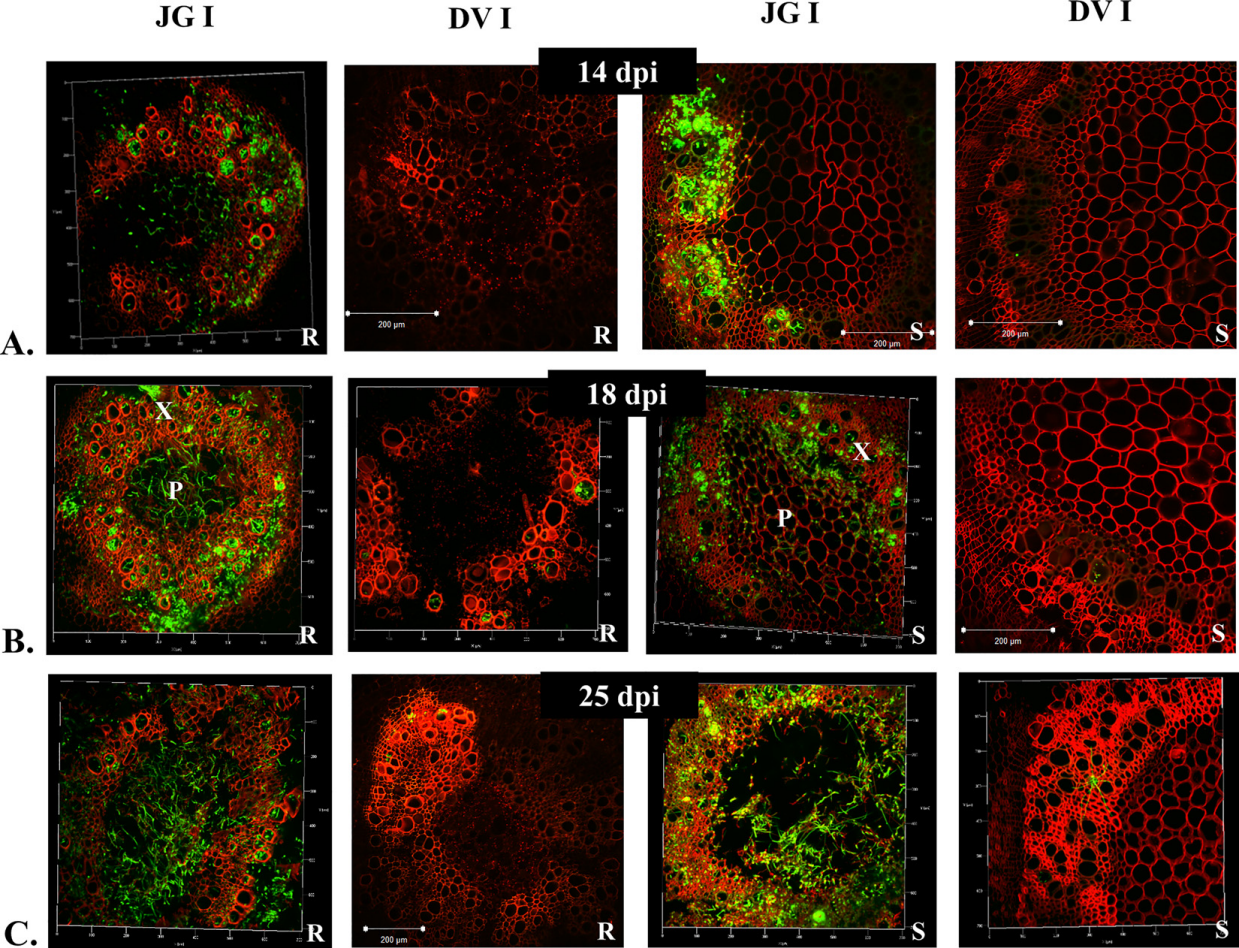
Temporal pattern of colonization of Foc 2 in both susceptible (JG62) and resistant (Digvijay) cultivars of chickpea. R- Root, S- Shoot, LR- Lower Root, MR- Middle Root, UR- Upper Root. **A.** (14 dpi) Transverse section of root and shoot reveals still higher fungal mass in JGI while nearly absence of fungus in DVI.(slight fluorescence marked in the figure). **B.** (18 dpi) Transverse section of root and shoot showing vasculature completely flooded with fungal mass in JGI and very few fungal hyphae in DVI. **C.** (25 dpi) Transverse section of root and shoot reveal distortion of vasculature because of heavy fungal colonization in JGI whereas plant tissues are near normal in DVI with little fungus here and there.

**Fig 4:**
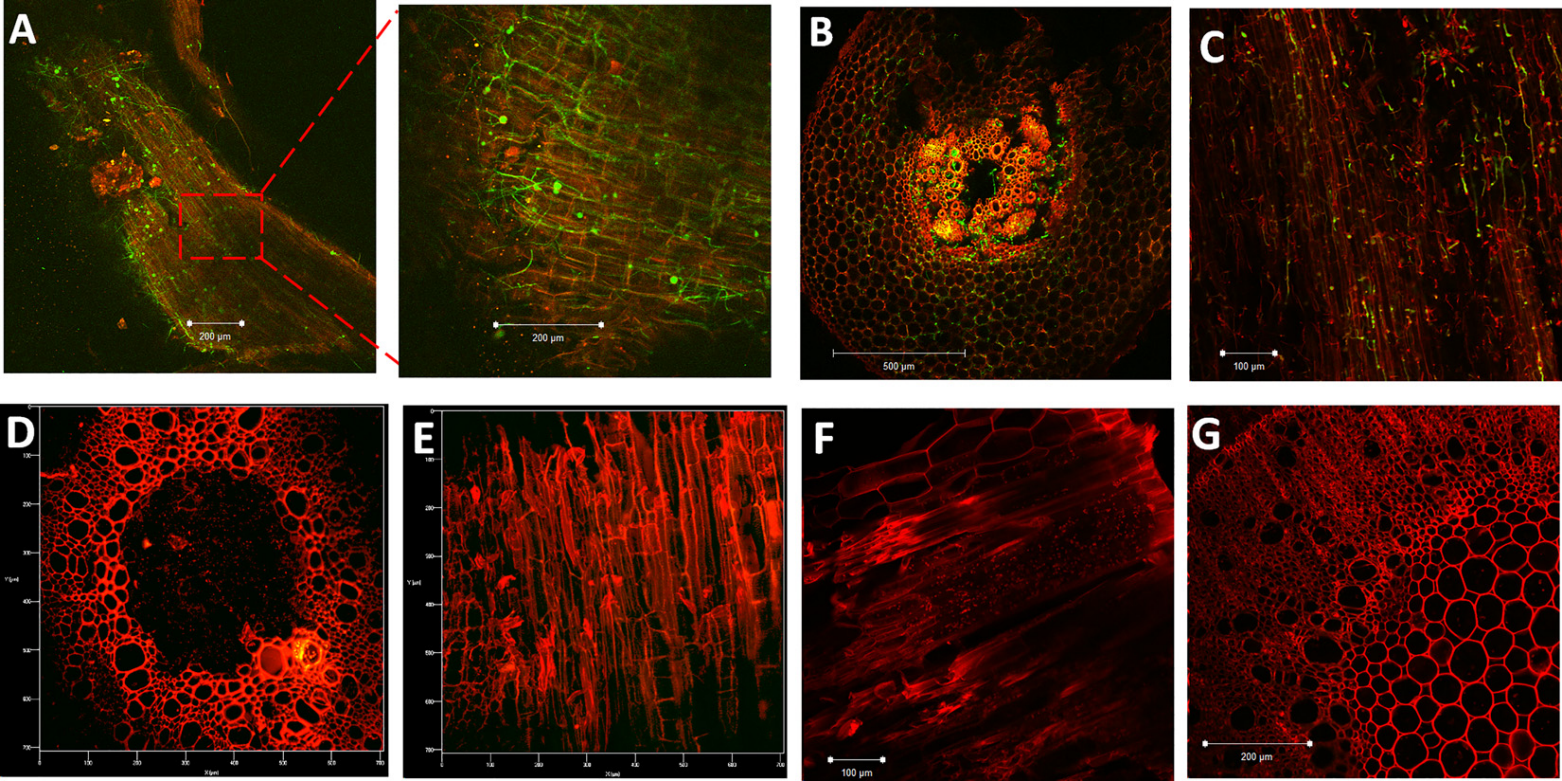
Late stages of chickpea root and shoot colonization by *Fusarium oxysporum* f.sp. *ciceri* race 2 marked with eGFP in susceptible (JG62) and resistant (Digvijay) cultivars at 28 DPI. **A**. Heavy colonization of root of susceptible (JG62) plant in longitudinal section. **B.** Cross section of root of susceptible (JG62) plant showing complete colonization of fungus in cortex (C) as well as xylem vessels (X) and deformation of root architecture. **C.** Longitudinal section of stem of susceptible (JG62) plant with the extensive presence of conidia as well as mycelia of fungus. **D-E.** Cross and longitudinal sections of root of resistant plant (Digvijay) with absence of any fungus and normal architecture of roots. **F-G.** Longitudinal and cross section of stem of resistant (Digvijay) plant without any fungal infection.

Based on these pilot studies, in-depth analysis of pathogen infection in JGI and DVI was performed throughout the disease progression. During early infection stages (up to 4–6 dpi), both cultivars showed surface colonization and entry of the pathogen in lower roots (**Fig 2A and 2B**). However, substantial colonization of vascular region was thereafter observed in lower and middle root zone of only JGI at 8 dpi (**Fig 2C**). Further, the appearance of wilting symptoms in JGI was marked with heavy colonization of lower, middle and upper root zones along with the lower stem region at 10–12 dpi (**Fig 2D**). However in DVI, initially the pathogen was restricted to root cortex region (**Fig 2C and 2D**) and reached xylem vessels very late (by 18 dpi) and that too in very less numbers. In JGI the pathogen reached as far as the fifth internode by 14 dpi (**Fig 3A**). By 25 dpi, both the root and stem of JGI were heavily colonized resulting in disruption of normal architecture, complete wilting and death of most of the plants; while the root and shoot architectures of DVI plants were nearly normal (**Fig 3B and 3C**). As depicted in **Fig 4A–4C**, these differences between JGI and DVI further intensified by 28 dpi; where JGI showed exhaustive colonization of both root and shoot tissues, while minimal fungal colonization was observed in DVI (**Fig 4D–4G**).

### *In planta* pathogen quantification using qPCR

#### Whole root analysis

**Fig 5:**
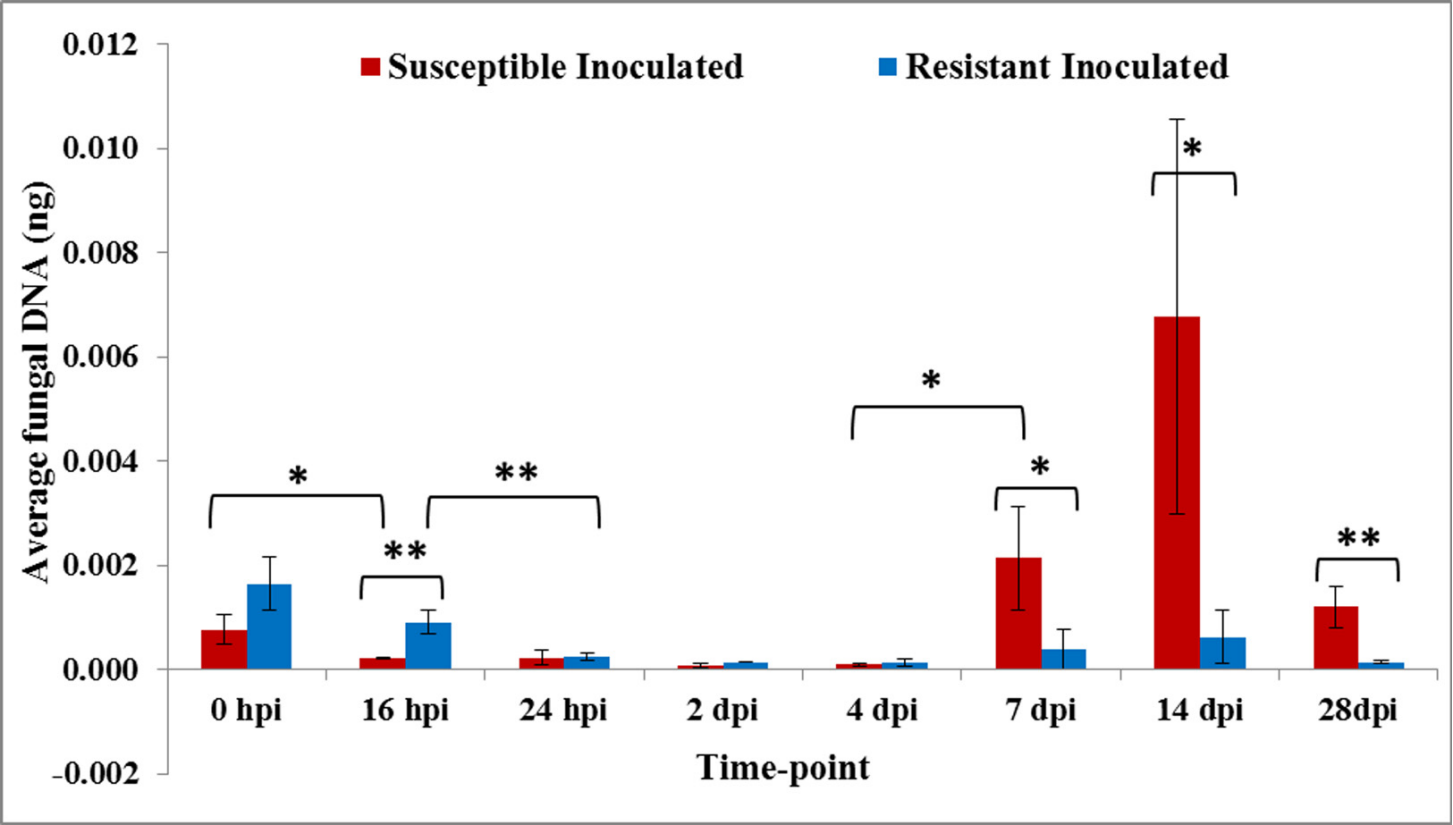
Proportion of Foc 2 DNA in whole roots of chickpea cultivars, JG62 (susceptible) and Digvijay (resistant), at different time-points after inoculation. Amount of pathogen DNA was estimated using quantitative polymerase chain reaction (qPCR). Bars with asterisk (*) indicate level of significance of the amount of pathogen between JGI and DVI as per T-test performed between the two groups. ‘*’- P<0.05; ‘**’- P<0.01; ‘***’-P<0.001.

A nearly perfect (R^2^ ~0.999) linear regression between the logarithm of known concentrations of fungal DNA and qPCR threshold cycles (Cts) was established (S2 Fig). Using these standard regression lines, significant amount of Foc 2 DNA was detected in both JGI and DVI till 16 hpi, followed by a decrease till 4 dpi. Thereafter, the amount of Foc 2 DNA increased significantly only in JGI till 14 dpi. At 28 dpi, the fungal DNA content decreased in both JGI and DVI; however in DVI, the pathogen load itself was significantly less than that in JGI (Fig 5).

#### Progressive colonization of Foc 2 in chickpea root and shoot fractions

**Fig 6:**
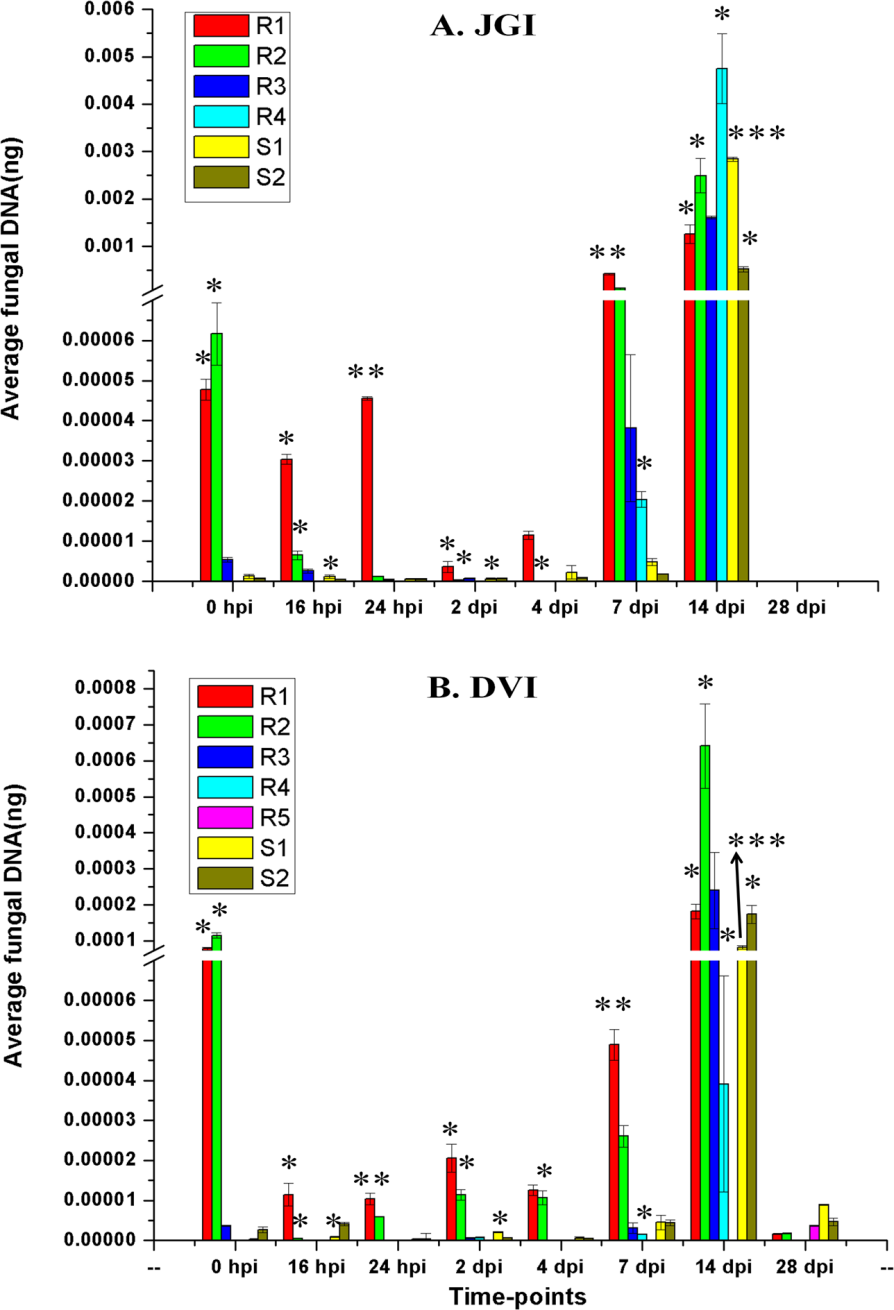
**Proportion of Foc 2 DNA in fractions of roots and shoots from the inoculated root tip of (A) susceptible (JG62) and (B) resistant (Digvijay) chickpea cultivars at various time-points after inoculation.** In case of JG62, 28 DPI root fractions could not be collected as the plant was completely wilted. Bars with asterisk (*) indicate level of significance of the amount of pathogen between JGI and DVI as per T-test performed between the two groups. ‘*’- P<0.05; ‘**’- P<0.01; ‘***’-P<0.001.

Genomic DNA was isolated from root (R1-R4 in JGI and R1-R5 in DVI) and shoot fractions (S1 and S2 in both JGI and DVI) to study the pathogen progression in JGI and DVI plants (S6 Fig), using qPCR based pathogen DNA quantification described before. The pathogen DNA detected at 0 hpi in R1 and R2 fractions of both JGI and DVI was higher compared to the subsequent time points till 4 dpi in JGI and 7 dpi in DVI (Fig 6A and 6B). At later time points (16 hpi to 14 dpi), the pathogen DNA increased gradually in DVI in R1 and R2 fractions, which reached the maxima at 14 dpi (Fig 6B). At this time point, the maximum amount of pathogen DNA was detected in all the four (R1-R4) root fractions and both (S1-S2) the shoot fractions. However at 28 dpi, the pathogen load significantly decreased in all the fractions. Interestingly, the pathogen load was below the detectable limit in R3 and R4 fractions at 28 dpi in DVI. The fraction R5 could be assigned only at this time point in DVI, as the root-length increased due to growth, and showed minor amount of pathogen DNA. In JGI, pathogen load remained high in R1 till 24 hpi and dipped during 2 to 4 dpi. Thereafter, pathogen DNA was detected in the entire root and shoot fractions with maximum at 14 dpi. All the root fractions of JGI had nearly 10 times higher pathogen load compared to that of DVI (Fig 6). In JGI, the 28 dpi root tissue was completely wilted and fragmentation was not possible.

### Expression of pathogen virulence related genes in chickpea

**Fig 7:**
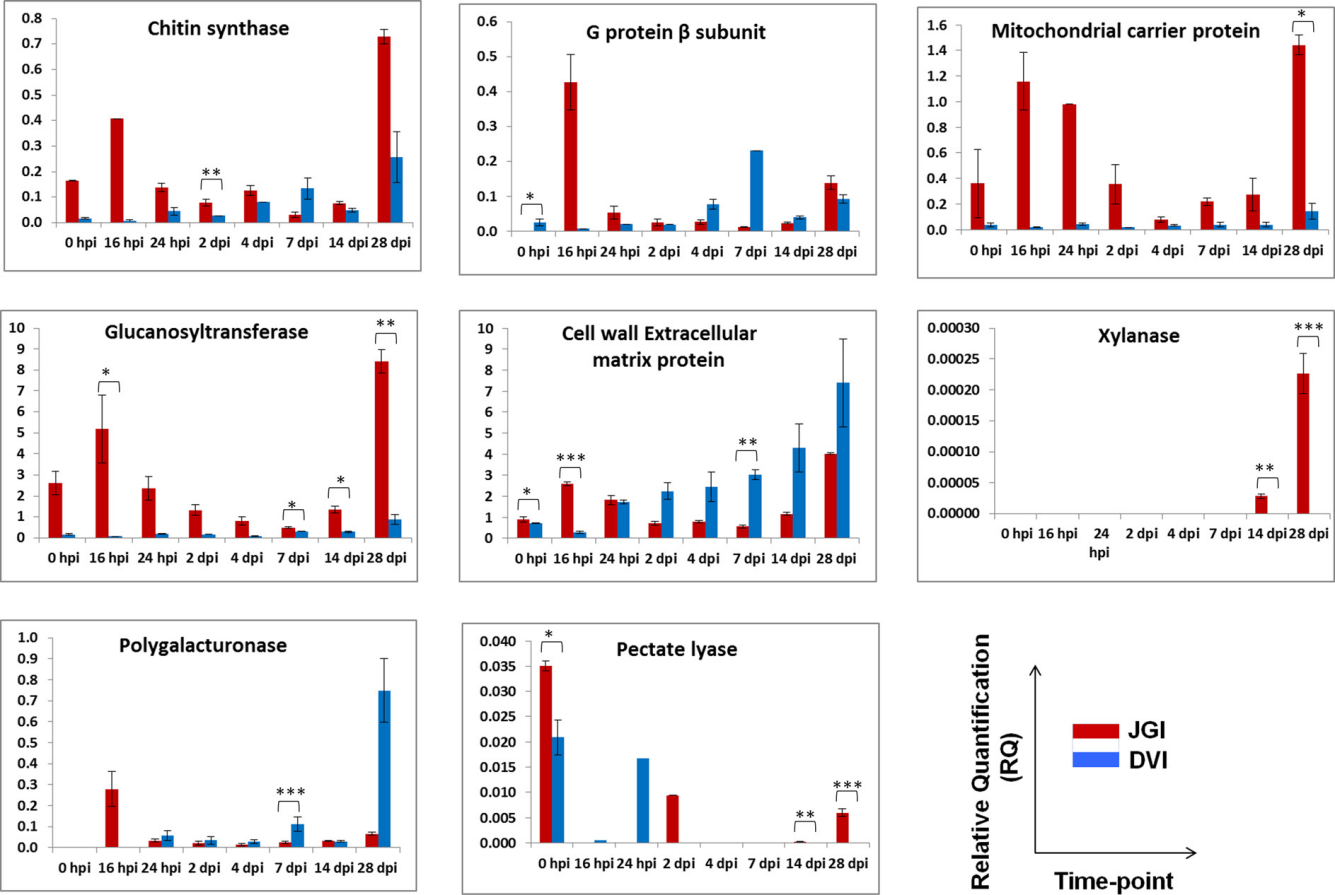
Expression patterns of eight fungal virulence related genes in JGI and DVI at eight different time-points using quantitative reverse transcriptase PCR. Bars with asterisk (*) indicate level of significance of the expression of particular gene between JGI and DVI as per the Student’s ‘*t*’ test performed between the two groups. ‘*’- P<0.05; ‘**’- P<0.01; ‘***’-P<0.001.

The expression of several pathogen virulence related genes in JGI and DVI was analyzed using qRT-PCR; however, only few of them could be successfully traced throughout the complete time-scale of infection (**Fig 7**). The expression of chitin synthase VII (*Chs7*), a chaperonin like ER protein was initially weak in JGI and DVI; however in JGI, the expression increased at 16 hpi followed by decrease and again increase at 28 dpi. In DVI, it was elevated at later stages reaching the maxima at 28 dpi. However, the expression was much higher in JGI compared to that in DVI at 28 dpi. The expression of G protein β subunit gene was very high at 16 hpi in JGI, which decreased with disease progression and again increased at 28 dpi. In DVI, the expression was initially weak and was elevated at 7 dpi. We found that the expression of this gene was very high in JGI at early stage of colonization, which can be correlated with hyphal growth and establishment. Similarly, the expression of mitochondrial carrier protein (*Fow1*), which is responsible for the transfer of tricarboxylates across the mitochondrial inner membrane, was initially high in JGI, decreased till 4 dpi, and thereafter gradually increased reaching its peak at 28 dpi. In DVI, the expression was almost nil with only weak expression at 28 dpi.

Glucanosyltransferases are the key enzymes involved in fungal cell wall synthesis and required for fungal growth and morphogenesis. The expression of glucanosyltransferase gene was high till 16 hpi, then decreased and again increased when plants showed wilting symptoms in JGI. While in DVI, the expression was very low throughout. Cell wall extracellular matrix proteins (CWEMPs) are the glycoproteins covalently linked to cell wall matrix and are major pathogen virulence factors. The expression of the CWEMP showed gradual increase with disease progression in DVI; whereas in JGI, initial increase in the expression of the gene followed a gradual decrease and the final elevation at end of the infection. Production of cell wall degrading enzymes (CWDEs) like xylanases (XYL), polygalacturonases (PG) and pectate lyases (PL) is vital for pathogen establishment in plants. We observed high expression of *XLY* gene only in JGI, particularly in late stages; while the expression was not detected in DVI. On the other hand, we found elevated expression of PG in initial hours of disease progression in JGI, which then decreased further. However, gene expression was evident only at 28 dpi in DVI. The expression of *PL* gene was higher at the start of infection in both JGI and DVI, which then declined at 16 hpi and again increased in DVI at 24 hpi and in JGI at 2 dpi. Thus the gene mainly expressed during initial surface colonization and up to 2 dpi, when the fungus invades root cortex; and vascular region thereafter in JGI, as also observed by confocal microscopy.

## Discussion

Plant-microbe interaction studies have been revolutionized by the high throughput ‘Omics’ methodologies. However, specific aspects of plant-pathogen interaction such as pathogen entry, localization and colonization in the host; as well as spatial and temporal behavior of the pathogen in compatible and incompatible reactions are not answered by these methods [34]. Such aspects could be understood using Confocal Laser Scanning microscopy (CLSM) and targeted gene expression analysis using qRT-PCR. The present study employed CLSM along with fungal mass estimation using qPCR to study the infection process of Foc 2, a highly virulent race of *Fusarium oxysporum* f.sp. *ciceri* active in Indian peninsula.

### Differential invasion of Foc in resistant and susceptible chickpea cultivars

To understand the possible mechanism of invasion of Foc in susceptible and resistant chickpea cultivars, transformation of the pathogen and its localization *in planta* paired with quantification in various plant tissues were performed. The five *eGFP* transformed Foc isolates did not show any altered phenotypic or virulence characteristics compared to the wild type; however, variation in GFP fluorescence was observed. The transformant D4 having the highest and uniform GFP fluorescence and virulence comparable to that of wild type was selected for studying the *in planta* pathogen progression using CLSM. We observed attachment and germination of fungal spores on to epidermal cells within 3 dpi followed by fast penetration of root epidermis, cortex and xylem of the susceptible cultivar by 4 dpi. These processes were, however, impeded in the resistant cultivar. Further, no specialized structure during penetration of Foc 2 in plant epidermis was observed. It was simply done by growing hyphal branch but not the germ tube. These findings are in accordance with previous studies in case of *Fusarium oxysporum* f.sp. *radicis-lycopersici* [25], *Fusarium oxysporum* f.sp. *melonis* [35] and *Fusarium oxysporum* f.sp. *fragariae* [36]. Interestingly, swellings were seen at penetration site of the penetrating hypha in the susceptible cultivar. This observation is also consistent with previous reports on other formae speciales of *F*. *oxysporum* in case of tomato and strawberry [25, 36, 37].

The transformed Foc 2 could be detected throughout the inspected plant parts during disease progression in susceptible inoculated cultivar (JGI), particularly with increasing fungal load. However, the pathogen could be seen only in root cortex region of inoculated resistant cultivar with very few mycelia escaping to vascular tissue. Similar colonization patterns for Foc races 0 and 5 in compatible and incompatible interactions in chickpea have been reported earlier [27]. Likewise, it has been demonstrated that *F*. *oxysporum* f.sp. *fragariae* was confined in the epidermal layer of roots in the resistant strawberry cultivar [36]. Even in resistant pea cultivar, *F*. *oxysporum* f.sp. *pisi* was restricted to the initially infected root vessels in asymptomatic reactions [35]. These differences of the colonization in the susceptible and resistant cultivars could be correlated with the differential defense mechanisms harbored in the chickpea genotypes as highlighted in our previous studies [7, 38].

### Probable phases of Foc 2 proliferation in the susceptible cultivar

Further, quantification of fungal colonization in various tissues of the resistant and susceptible chickpea cultivars was performed by qPCR. Based on this, four distinct phases of fungal proliferation can be put forth. In phase 1 (0 hpi) high amount of fungal DNA was observed in both the cultivars, indicating adherence and germination of the fungal spores. Phase 2 (16 hpi—4 dpi) was marked by the decrease in the fungal DNA suggesting the degradation of a fraction of the hyphae due to autophagy. Similar phenomenon was reported in *F*. *graminearum* during colonization in wheat [39]. This could be attributed to the fact that, before the pathogen colonizes the plant to the extent that it can derive nutrients from the host, it undergoes intracellular degradation to supply nutrients to the non-assimilating fungal structures. Steep increase in fungal DNA was observed in phase 3 (4 dpi—14 dpi) indicating widespread colonization of the fungus in JGI compared to that in DVI. During this phase, JGI also showed the typical wilting symptoms like chlorosis, drooping of petioles etc. Lastly, the phase 4 (14 dpi—28 dpi) was marked by decrease in fungal DNA content in JGI (**Fig 5**). This indicated that the fungus proliferated massively in JGI till nutrients from the host were available (phase 3), resulting in high mycelial mass and pathogen DNA. After the nutrients from the host were exhausted (due to wilting), the pathogen switched to conidiation, leading to reduced mycelial mass and as a result, pathogen DNA. Alternatively in DVI, successful activation of defense responses early in the infection process (before phase 3) might have restricted the fungal proliferation throughout the course of infection.

### Gene expression patterns support pathogen proliferation phases in the host plant

Successful infection of the pathogen to the host plant requires a number of important steps like recognition and adhesion to host tissue, degradation of host tissue and resistance to host antimicrobials etc. In the present study, some of the genes involved in these processes were analyzed by qRT-PCR. It is known that hyphae of phytopathogenic fungi navigate the host surface topography for identifying the vulnerable sites of invasion where they mechanically penetrate by expansion of growing hyphal tip [40, 41]. Furthermore, fungal hyphae are predicted to resist opposing forces at their tips during such penetration [34]. Thus fungal morphogenesis is an essential component of host invasion [42]. Chitin is considered as a structurally important component of fungal cell walls and chitin synthases, the enzymes implicated in chitin synthesis, belonging to different divisions and classes are found in fungi. In the present study, chitin synthase 7 (*Chs7*) was preferentially expressed in the JGI suggesting strong defense response in DVI. Our results correlated well with the earlier reports wherein chitin synthases are reported to be essential for virulence and invasive growth during plant infection in fungi like *Magnaporthe grisea* [43] and *Fusarium oxysporum* [44].

As communications between the pathogen and the plant are critical for disease development, signaling pathways that mediate these communications are also important. Several proteins like G proteins, MAP kinases, protein kinases A are known to be involved in such pathways and studies have shown their importance in fungal development and virulence [45–49]. Our results were in accordance with the above mentioned studies where G protein β subunit expression was the least in DVI in which pathogen could not establish the infection. Mitochondrial carrier proteins (MCPs) are small transport proteins of the mitochondrial inner membrane that catalyze the transport of metabolites across the inner membrane with a high degree of substrate specificity [50–52]. The present study also revealed the expression of MCP *Fow1* preferentially in JGI. This highlights the importance of this gene during establishment in the host. Our findings correlate with earlier reports where *Fow*1 was shown to be essential for colonization during infection [7, 53].

We also detected higher expression of the enzyme glucanosyltransferase, which is essential for fungal morphogenesis, and reported to be required for virulence [54, 55]. The present study validated the expression of these genes [7] using qRT-PCR over a wider range of duration, from 0 hpi to 28 dpi. Apart from these genes, several others were observed for their expression in the present study. Cell wall glycoproteins of the fungus are involved in species specific adhesion processes and also increase resistance of fungi to antimicrobial proteins produced by plants [56, 57]. Interestingly, the expression pattern of the cell wall extracellular matrix protein (CWEMP) showed gradual increase in resistant cultivar upon inoculation. This might be due to the pathogen making constant attempts to establish itself in the resistant cultivar, while protecting itself from the strong defense response of the resistant cultivar. In JGI however, initial increase in CWEMP expression accounted for successful establishment of the pathogen in the plant, followed by a transient decrease, which corresponded to phase 2; i.e. autophagy of the fungus for its own growth. When the fungus attains sufficient biomass to derive nutrients from the plant, the increase in CWEMP expression again indicates the attempts of the pathogen to colonize newer plant tissues.

Phytopathogenic fungi produce an array of extracellular hydrolytic cell wall degrading enzymes (CWDE) that enable them to penetrate and infect the host tissue. Plant cell wall degradation is essential in pathogenesis owing to the fact that the pathogen invades epidermis and grows through cortex to finally reach at xylem [11]. Therefore, the expression of CWDEs like PG, PL and XLY was evaluated in the present study. The expression of XLY was detected only in JGI and only at late stage of disease, when the pathogen entered the necrotrophic phase. In this phase, it probably played role in plant cell wall degradation. Our results are in accordance with the published reports indicating increased XLY activity with the disease progression [11] and contribution of XLY to the infection process by inducing necrosis of the infected plant tissue [58].

Interestingly, PG expressed initially only in JGI and its expression decreased with the disease progression and symptom development in JGI, in accordance with the results reported previously [11]. On the contrary, the expression of PG was detected only at late stages in DVI. These enzymes loosen the pectin network in plant cell wall and help the fungi to secrete the digestive enzymes for nutrient acquisition. Thus, their expression in late stages in resistant plants suggests attempts of the fungus to acquire nutrients in growth limiting environment, where the fungus is present in minimal number owing to the strong defense response of the resistant host. Furthermore, as evidenced by confocal studies, plant architecture of DVI remained almost normal throughout the disease progression, due to which intact pectin of the plant cell wall might have induced the production of PGs. Such expression of PGs in nutrient depriving condition as well as in the presence of pectin has been reported earlier [59, 60]. Another CWDE, PL was found to express at three time-points covering initial invasion and colonization, invasion from cortical cells to xylem and necrotic phases. Along with PG, PL has also been postulated to be involved in plant penetration and colonization by phytopathogens [61]. Our results are in accordance with the study depicting abundant expression of PL gene early in the infection process and required for full virulence in *Alternaria brassicicola* [62].

The expression patterns of the candidate virulence genes analyzed in this study correlate well with colonization pattern observed in the whole root and root-shoot fractions by qPCR. The genes like chitin synthase, glucanosyltransferase, G protein β subunit and mitochondrial carrier protein, which play important role in fungal growth and morphogenesis, were expressed significantly during the initial colonization period, when the pathogen tried to establish in the host environment. This was followed by the decrease in expression of these genes pertaining to autophagy phase. The last phase was again the rise in expression revealing successful invasion and further proliferation of pathogen in the susceptible host. This rise was significantly high in JGI compared to that in DVI as the pathogen could colonize to great extent only in JGI.

## Conclusions

In summary, we highlighted the colonization process of Foc 2 in both, wilt-resistant and wilt-susceptible, chickpea cultivars using microscopic visualization of *eGFP* transformed Foc 2 as well as qPCR. Foc 2 colonized JG62, the wilt-susceptible cultivar profusely, while it was restricted in the root cortex in Digvijay, the wilt-resistant cultivar. Likewise, the pathogen could successfully express the genes essential for its entry, establishment and colonization in the susceptible plants. However, in resistant plants, the expression of most of these genes was severely limited. Based on these observations, it can be suggested that resistant plants were able to detect the pathogen early, activate defense response and restrict it in the cortex region; whereas, susceptible plants could not defend the pathogen early enough and the pathogen quickly colonized the plant tissues, blocked the nutrient transfer and killed the plants. This study further leads to analyze targeted disruption, RNAi mediated inhibition and overexpression of specific genes to confirm the function of these virulence genes.

## Supporting Information

S1 Fig The scheme of preparation of a binary vector modified pCAMBIA 1302. The mGFP cassette from pCAMBIA 1302 was replaced with eGFP cassette from pCBdeltaXCE using restriction enzymes *Xba*I and *Bst*EII.
(TIF)

S2 Fig Standard regression lines of three replicates of 10-fold serial dilution of Foc 2 DNA (10 ng/μl). Threshold cycles (Ct) were plotted against the log of known concentrations of Foc 2 genomic DNA.
(TIF)

S3 Fig A gel picture of semi-quantitative PCR of internal control *EF1α* in Foc 2 challenged JG 62. cDNA of all time-points was PCR amplified using *EF1α* specific primers in triplicates.
(TIF)

S4 Fig Phenotypic changes in chickpea cultivars, JG62 and Digvijay, un-inoculated control (JGC & DVC) and Foc 2 inoculated (JGI & DVI), 14 days and 28 days after inoculation (DPI). Red arrows indicate typical wilting symptoms in susceptible inoculated cultivar (JGI).
(TIF)

S5 Fig PCR amplification of wild type and D4 transformant of Foc 2. Lane 1- wild type Foc 2 DNA with no amplification, Lane 2- D4 DNA with *hph* amplification (495 bp), Lane 3- wild type Foc 2 DNA with no amplification, Lane 4- D4 DNA with *eGFP* amplification (546 bp), Lane 5- wild type Foc 2 DNA with no amplification, Lane 6- D4 DNA with *hph* amplification (1007 bp), Lane 7–100 bp ladder.
(TIF)

S6 Fig Schematic presentation of root and shoot fractions used for estimation of Foc 2 using qPCR. Root fractions marked from R1 to R5 and shoot fractions S1 and S2. 2 inch fraction each from both cultivars were used for quantitative estimation of Foc 2.
(TIF)
