# Supplementary figures and images for: Dynamics of Colonization and Expression of Pathogenicity Related Genes in Fusarium oxysporum f.sp. ciceri during Chickpea Vascular Wilt Disease Progression
Source: PLoS One. 2016 May 26;11(5):e0156490. doi: 10.1371/journal.pone.0156490 (PMC4882060; doi:10.1371/journal.pone.0156490)

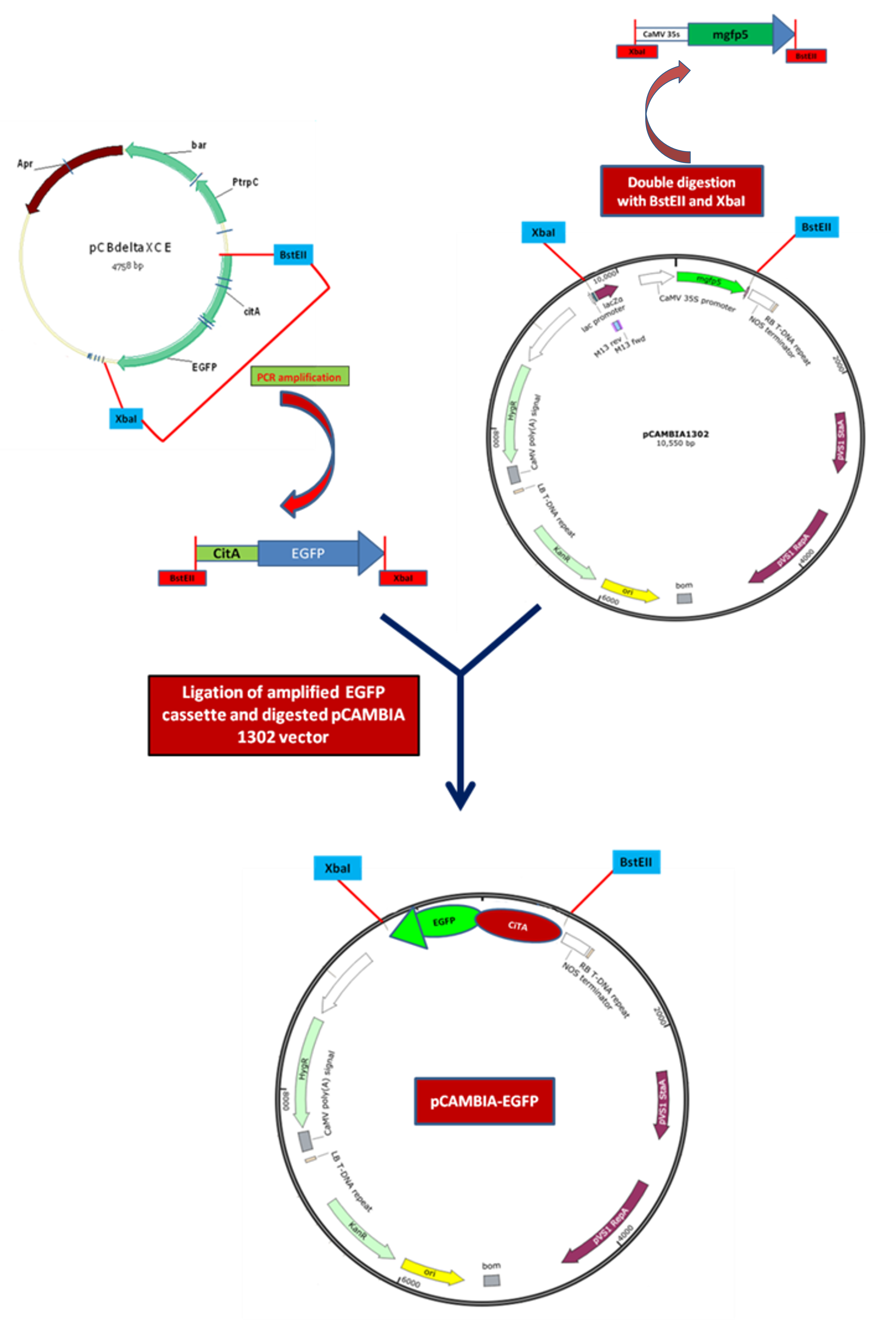

Supplement: S1 Fig — The mGFP cassette from pCAMBIA 1302 was replaced with eGFP cassette from pCBdeltaXCE using restriction enzymes XbaI and BstEII. (TIF) [file pone.0156490.s001.tif]

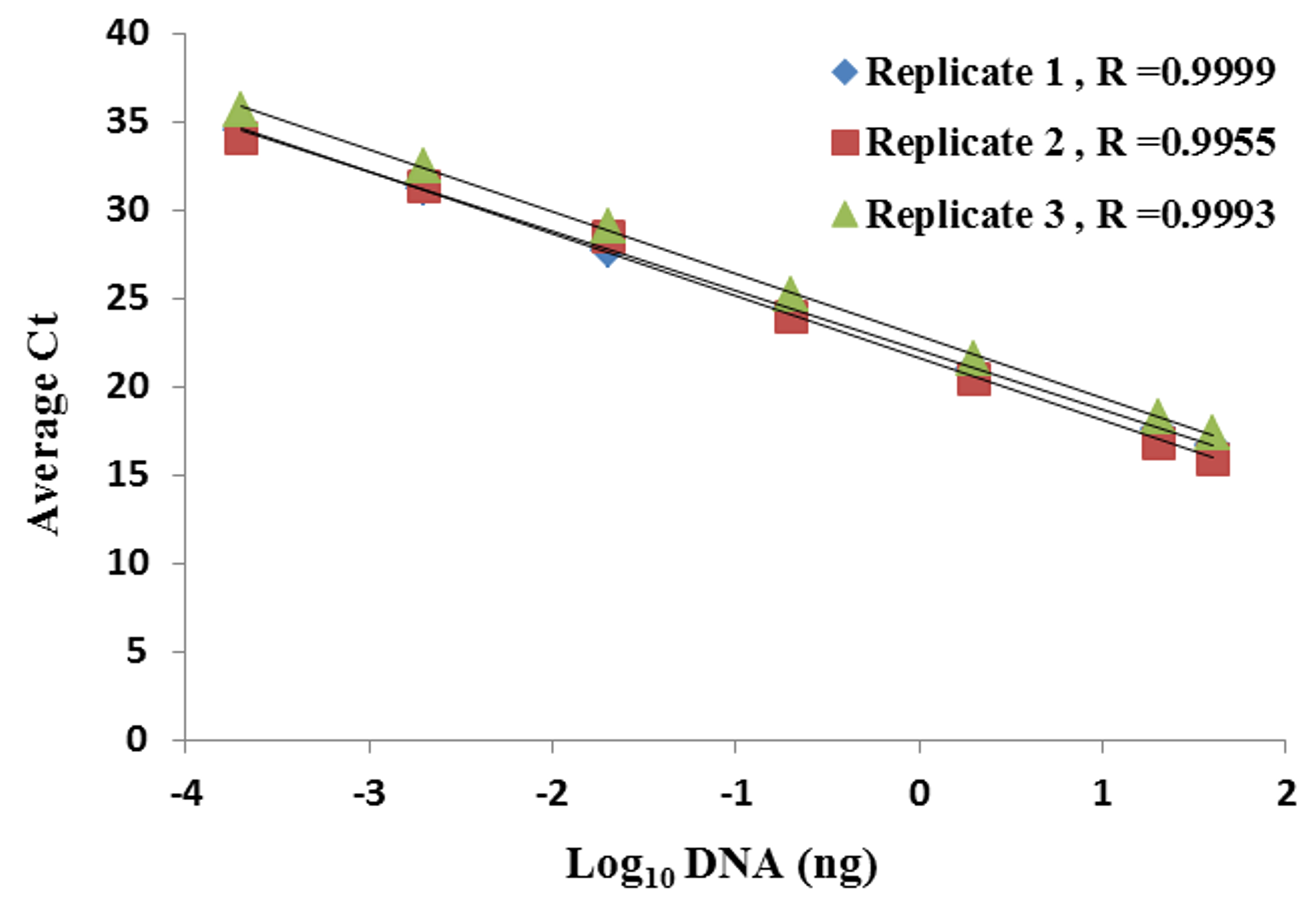

Supplement: S2 Fig — Threshold cycles (Ct) were plotted against the log of known concentrations of Foc 2 genomic DNA. (TIF) [file pone.0156490.s002.tif]

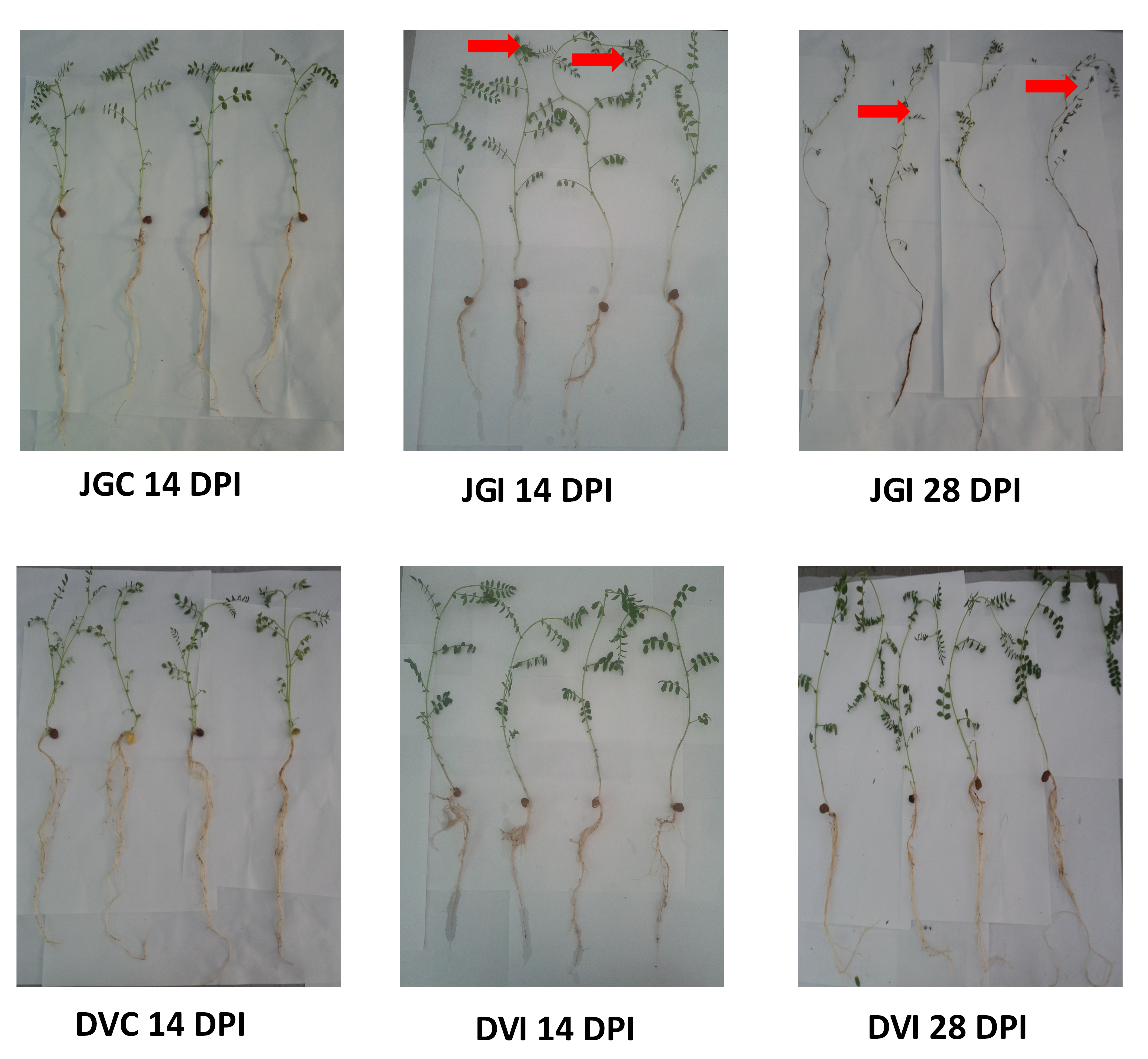

Supplement: S4 Fig — Red arrows indicate typical wilting symptoms in susceptible inoculated cultivar (JGI). (TIF) [file pone.0156490.s004.tif]

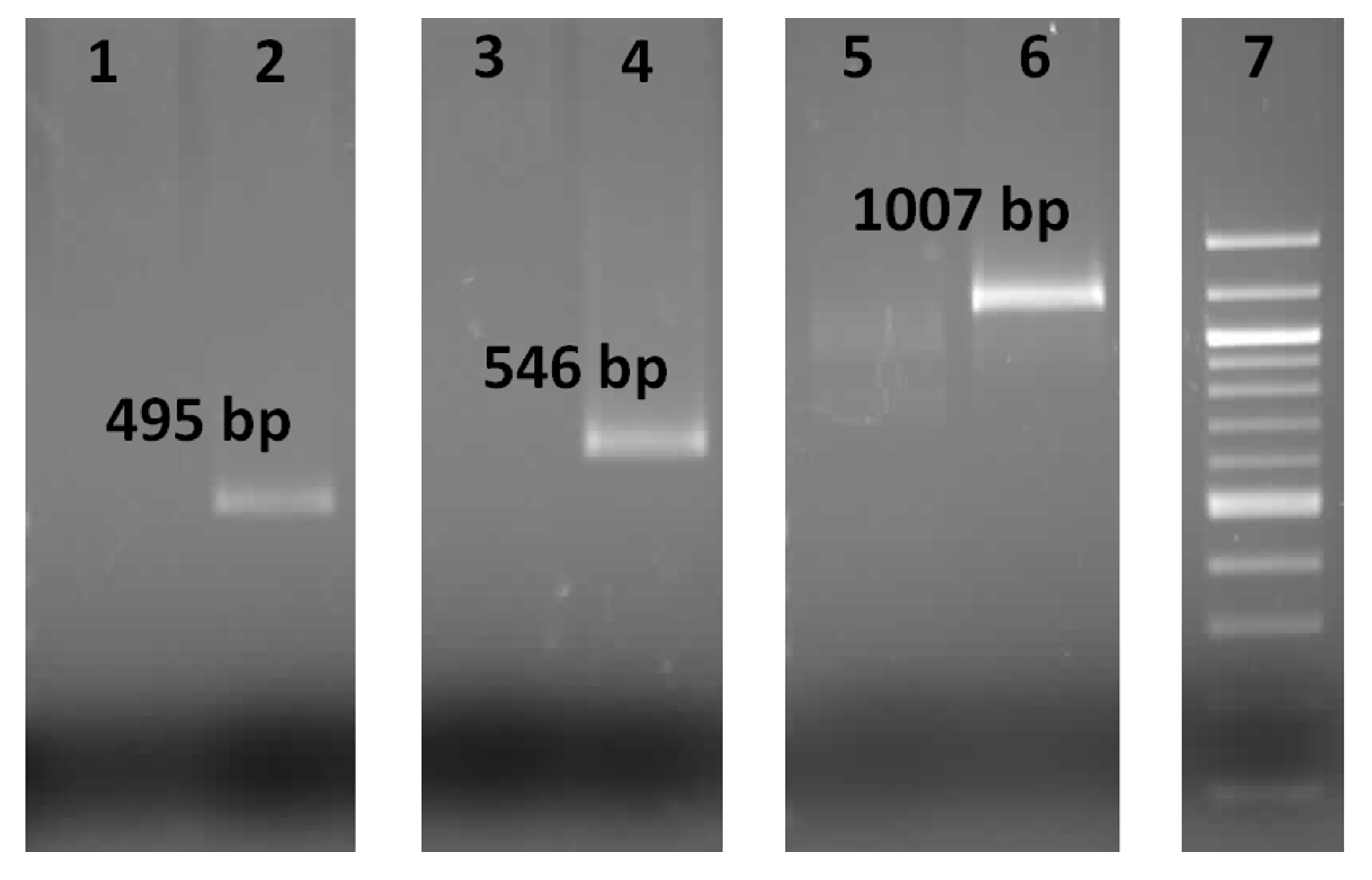

Supplement: S5 Fig — Lane 1- wild type Foc 2 DNA with no amplification, Lane 2- D4 DNA with hph amplification (495 bp), Lane 3- wild type Foc 2 DNA with no amplification, Lane 4- D4 DNA with eGFP amplification (546 bp), Lane 5- wild type Foc 2 DNA with no amplification, Lane 6- D4 DNA with hph amplification (1007 bp), Lane 7–100 bp ladder. (TIF) [file pone.0156490.s005.tif]

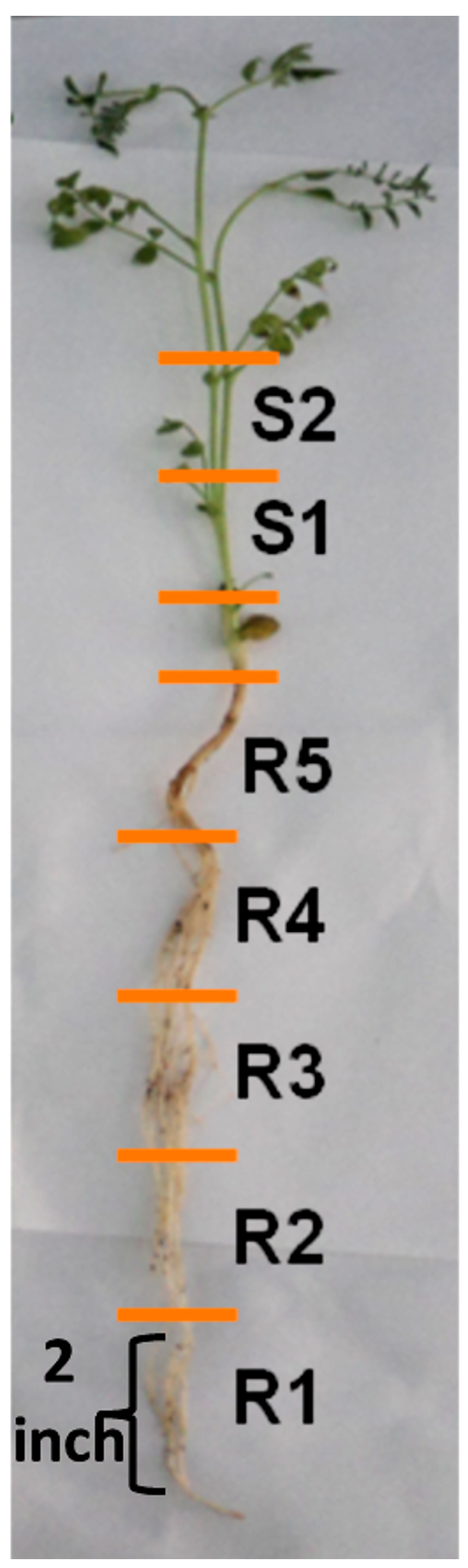

Supplement: S6 Fig — Root fractions marked from R1 to R5 and shoot fractions S1 and S2. 2 inch fraction each from both cultivars were used for quantitative estimation of Foc 2. (TIF) [file pone.0156490.s006.tif]
